# A High Through-Put Screen for Small Molecules Modulating MCM2 Phosphorylation Identifies Ryuvidine as an Inducer of the DNA Damage Response

**DOI:** 10.1371/journal.pone.0098891

**Published:** 2014-06-05

**Authors:** Jennifer FitzGerald, Laura S. Murillo, Gemma O'Brien, Enda O'Connell, Aisling O'Connor, Kevin Wu, Guan-Nan Wang, Michael D. Rainey, Alessandro Natoni, Sandra Healy, Michael O'Dwyer, Corrado Santocanale

**Affiliations:** 1 Centre for Chromosome Biology and National Centre for Biomedical Engineering Science, School of Natural Sciences, National University of Ireland Galway, Galway, Ireland; 2 School of Medicine, National University of Ireland Galway, Galway, Ireland; 3 Screening Core, National Centre for Biomedical Engineering Science, Biosciences, National University of Ireland Galway, Galway, Ireland; Universita' di Milano, Italy

## Abstract

DNA replication is an essential process for cell division and as such it is a process that is directly targeted by several anticancer drugs. CDC7 plays an essential role in the activation of replication origins and has recently been proposed as a novel target for drug discovery. The MCM DNA helicase complex (MCM2-7) is a key target of the CDC7 kinase, and MCM phosphorylation status at specific sites is a reliable biomarker of CDC7 cellular activity. In this work we describe a cell-based assay that utilizes the “In Cell Western Technique” (ICW) to identify compounds that affect cellular CDC7 activity. By screening a library of approved drugs and kinase inhibitors we found several compounds that can affect CDC7-dependent phosphorylation of MCM2 in HeLa cells. Among these, Mitoxantrone, a topoisomerase inhibitor, and Ryuvidine, previously described as a CDK4 inhibitor, cause a reduction in phosphorylated MCM2 levels and a sudden blockade of DNA synthesis that is accompanied by an ATM-dependent checkpoint response. This study sheds light on the previously observed cytotoxity of Ryuvidine, strongly suggesting that it is related to its effect of causing DNA damage.

## Introduction

The replication of the DNA is a fundamental process for cell division. Its execution is tightly controlled so that the genetic information is faithfully transmitted from mother to daughter cells. CDC7 kinase has key functions in this process; its most studied role is to promote the initiation of DNA replication by activating the replicative DNA helicase (MCM complex) bound at origins [1], [2]. Human CDC7 has also been shown to be involved in regulating the cellular response to DNA replication stress by 1) promoting DNA translesion synthesis [3] and 2) phosphorylating Claspin and promoting the first steps of the checkpoint response [4], [5].

At replication origins CDC7 phosphorylates several subunits of the MCM complex including MCM2-4-6. MCM phosphorylation by CDC7 is required for the recruitment of several other replication factors leading to the formation of active replication forks. In budding yeast phosphorylation of MCM4 was shown to relieve an inhibitory effect on helicase activity [6]. To date the specific consequences of MCM2 phosphorylation are not clear, although it has been proposed that it is important for MCM loading onto replication origins in the cells reentering the cell cycle [7]. CDC7 phosphorylation of human MCM2 occurs at several sites, and biochemically CDC7 has a preference for serines that are followed by negatively charged groups such as acidic amino acids or phosphorylated serines and threonines [5], [8], [9]. In particular Ser40 phosphorylation only occurs when Ser41 is also phosphorylated by a different kinase, that acts as a priming event [9]. During the cell cycle, MCM2 Ser41 phosphorylation is constitutive while phosphorylation on Ser40 fluctuates in a manner that strictly correlates with CDC7 activity [9]. Furthermore, studies using siRNA-mediated downregulation of CDC7, as well as CDC7 kinase inhibition with a wide variety of small molecule inhibitors, have demonstrated that Ser40 MCM2 phosphorylation is a robust and reliable indicator/biomarker of cellular CDC7 activity [10], [11]. Intracellular CDC7 activity is regulated at multiple levels: by the binding of a regulatory subunit, either DBF4A or DBF4B [12]–[14], by cell cycle dependent transcription of the catalytic and regulatory subunits [15], by APC dependent proteolysis [16] and by miRNA's [17]. CDC7-dependent phosphorylation of MCM proteins is then antagonized by cellular protein phosphatases, with PP1 having a major role in this process in both budding yeast and Xenopus [18], [19].

The critical roles of CDC7 in promoting DNA replication and responding to DNA damage and replication stress have led to the development of small molecule CDC7 inhibitors, which have been shown to affect DNA synthesis as well as having cytotoxic activity and potent anticancer activity in preclinical cancer models [10], [11], [20]–[26]. However, it is well known that the development of new drugs takes enormous amounts of time, money and effort, with the translation of a promising molecule into an approved drug often taking more than 13 years [27]. It is therefore crucial to explore other strategies to reduce these limitations and drug “rescue” and “repurposing” provides such an opportunity [28]. Drug “rescue” identifies small molecules and biologics that can modulate the molecular function of the target of interest, which were previously developed in other unrelated studies (but not further developed or submitted for FDA approval). Instead “repurposing” generally refers to studying a small molecule or a biologic approved by the FDA to treat one disease or condition, to see if it is safe and effective for treating other diseases. The advantage of rescued and repurposed drugs is that detailed information is often available on their pharmacology, formulation and potential toxicity, and they may even have been tested in humans, which would considerably speed up their development for a new therapeutic indication [29].

Therefore, with the dual goal of obtaining insight into CDC7 regulation and identifying small molecules that affect CDC7 activity that could be eventually rescued or repurposed, we set up a cell-based assay capable of measuring levels of pSer40/41 MCM2 as readout of CDC7 activity. This assay was suitable for high-throughput screening and was used to screen the Johns Hopkins Clinical Compound Library which contains 1514 compounds of which 1082 are FDA approved drugs and 432 are foreign approved drugs and the Tocriscreen Kinase Inhibitor Toolbox which contains 80 kinase inhibitors.

In this work we report the identification of several FDA approved drugs capable of altering pSer40/41 MCM2 levels in HeLa cells and the characterization of Ryuvidine, a reported CDK4 inhibitor, as a novel DNA synthesis blocker.

## Material and Methods

### Cell culture

HeLa S3 (CCL-2.2) cells used for the screening and cell based assays were authenticated by LGC standards at the time of the screening [5], U2OS [30] and human foreskin fibroblasts (HFF) [31] cells were cultured in Dulbelcco's modified Eagle's medium (DMEM) supplemented with heat inactivated 10% fetal bovine serum and 1% penicillin-streptomycin (Sigma-Aldrich).

### Chemicals

Tocriscreen Kinase Inhibitor Toolbox (Cat. No. 3514), Ryuvidine (Cat. No. 2609), Flupenthixol dihydrochloride (Cat No. 4057), PHA767491 (Cat. No. 3140), were from Tocris Bioscience. ATM inhibitor KU55933 (Cat No. S1092) was from Selleckchem. The Johns Hopkins Clinical Compound Library (JHCCL) was obtained from Johns Hopkins School of Public Health. Pimagedine (Cat. No. 109266), Primaquine biphosphate (Cat. No. 160396), Bornyl Acetate 95% (Cat. No. B55203), Ticarcillin disodium salt (Cat. No. T5639), Mitoxantrone dihydrochloride (Cat No. M6545), Quinacrine dihydrochloride (Cat. No. Q3251) were from Sigma-Aldrich Ltd. DRAQ5 was supplied by Biostatus Limited. Caspase inhibitor Boc-D-fmk (Cat No. 1160-5) was from Biovision.

### Antibodies, plasmids and peptides

Anti-pSer40/41MCM2 antibody was raised by GenScript (GenScript USA Inc) using a CAPLT(p)S(p)SPGR peptide and further affinity purified. Cleaved PARP (19F4) and cleaved Caspase 3 (5A1E) antibodies were from Cell Signalling Technology; CDC7 (clone SMP171) and β-tubulin (ab6046) antibodies were from Abcam; β-Actin antibody (Cat No. A00702) was from GenScript; MCM2 antibody (Cat No. MCA1859) was from AbD Serotec; pSer41MCM2 antibody (Cat no. A300-117A-1) was from Bethyl Laboratories Inc; γ-H2AX antibody, recognizing pSer139-H2AX (Cat No. 05-636) was from Millipore; pSer108MCM2 antibody was previously described [9]. Secondary antibodies labeled with infrared fluorophores (800CW anti-rabbit Cat No. 926-32211, 800CW anti-mouse Cat no. 926-32210, 680LT anti-rabbit Cat No. 926-68021 and anti-mouse Cat no. 926-68020) were obtained from Li-COR Biosciences Ltd, secondary antibodies labeled with TRITC fluorophore were obtained from Jackson ImmunoResearch. Odyssey Infrared Imaging Systems were from Li-COR Biosciences Ltd.

### Kinase, phosphatase and peptide competition assays

To perform in vitro kinase reactions, 2 µg of recombinant GST-MCM2aa 9-294 was incubated with 250 ng of CDC7/DBF4 in the presence of 1.4 µM ATP and 10 mM MgCl_2_ at pH 7.6 for 30 min at 30°C as previously described [9]. Reactions were then heated to 95°C for 3 minutes in Laemmli buffer and analyzed by western blot.

For peptide competition experiments, the pSer40/41MCM2 antibody was preincubated with either an unphosphorylated CAPLTSSPGR peptide, a monophosphorylated CAPLTS(p)SPGR peptide, or a double-phosphorylated CAPLT(p)S(p)SPGR peptide, for 1 h at room temperature before immunoblotting or immunohistochemistry. A 767.5 to 1 molar ratio peptide to antibody was used.

For the phosphatase assay, 20 µg of HeLa protein extracts were incubated for 30 minutes at 30°C in the presence of 400 U of λ-phosphatase (Sigma-Aldrich Ltd., Cat No. P9614).

### Immunoblotting

Whole cell lysates were prepared in TGN buffer (50 mM Tris-HCl, pH 7.5, 200 mM Sodium Chloride, 50 mM Sodium β-glycerophosphate, 50 mM Sodium Fluoride, 1% Tween-20, 0.02% NP40) containing protease and phosphatase inhibitors as previously described [32]. Protein concentration was determined using the Bradford reagent (Sigma), separated by SDS-PAGE and transferred to nitrocellulose membrane. Membranes were probed overnight at 4°C with relevant primary antibodies and with infrared-labeled secondary antibodies. Immunoreactive bands were visualized and quantified using Odyssey Infrared Imaging Systems (Li-Cor Biosciences).

### Immunofluorescence microscopy

For DNA synthesis studies HeLa cells growing on coverslips in a 6-well plate were treated with relevant drugs (as described in the text) and 15 minutes before fixation 10 µM EdU (Berry & Associates, PY 7562) was added. Cells were fixed with 4% paraformaldehyde for 15 minutes at room temperature. Cells were washed three times with PBS, then permeabilised by treating twice for 10 minutes with PBS-TX (PBS/0.1% Triton X-100) at room temperature. Click reaction mix (0.1 mM 6-carboxyfluorescein TEG-Azide (Berry & Associates, FF 6110), 10 mM Sodium-L-Ascorbate and 2 mM Copper-II-Sulphate) was added to the cells for 30 minutes in the dark. Coverslips were washed three times for 5 minutes with 1% BSA 0.5% Tween in PBS at room temperature in the dark to remove excess copper and azide. Nuclei were stained with DAPI and coverslips were mounted using SlowFade Gold Antifade Reagent (Invitrogen, S36936).

For pSer40/41MCM2 and γ^∼^H2AX staining cells were treated and fixed as above. Primary anti-pSer40/41MCM2 and anti-γ^∼^H2AX antibody (Millipore, 05-636) were used together with secondary Alexa Fluor 546 goat anti-rabbit antibody (Life Technologies, A11010) or Alexa Fluor 546 goat anti-mouse antibody (Life Technologies, A11003) respectively.

### Immunohistochemistry

The use of anonymized formalin fixed paraffin embedded tissue samples in this study was approved by the Clinical Research Ethics Committee, Merlin Park Hospital, Galway. The ethics committee of the institution waived the need of consent for the use of these archived samples. Serial consecutive sections of tissues (3.5 µm) in microarray format were dewaxed in xylene and rehydrated in graded ethanol to water. Heat-induced epitope retrieval was performed for 10 minutes in citrate buffer (pH 6.0). Endogenous peroxidase was blocked by treatment with 3% H_2_O_2_ in methanol for 20 minutes followed by 30 minutes blocking in PBS plus 1% (w/v) BSA and 2% (v/v) fetal calf serum. Sections were stained with 0.07 µg anti-pSer40/41MCM2 overnight at 4°C and washed with PBS the following day. Sections were then incubated in ImPress universal anti-mouse/anti-rabbit antibody reagent (Vector Laboratories, Burlingame, CA) for 30 minutes, developed using ImmPACT DAB peroxidase substrate (Vector Laboratories) and counterstained with Hematoxylin. This was followed by dehydration with graded ethanol, cleared with HistoClear and coverslips mounted with DPX. Incubation without primary antibody was used as a negative control. Pictures were taken with an Olympus BX61 microscope using Cell Soft Imaging Software (Olympus UK Ltd).

### In-Cell Western Screening

HeLa cells were seeded at 20,000 cells in a volume of 90 µl per well in 96-well plates and grown for 20 hours in 5% CO_2_ at 37°C to achieve 70%–80% confluency. Fresh dilutions of the drugs were prepared in warm cell culture media from the mother plates and added directly to each well at a concentration of 10 µM. After nine hours of incubation cells were washed with PBS and fixed with 3.7% paraformaldehyde in PBS for 20 minutes. Cells were then permeabilised by five washes with Triton X-100 in PBS (5 minutes each) and incubated with PBS 1% (w/v) BSA and 2% (v/v) fetal calf serum for 90 minutes at room temperature. Primary antibody was diluted in blocking solution, added to the cells to a final concentration of 0.8 µg/ml and then incubated overnight at 4°C. On the following day cells were washed six times with 1% Tween-20 in PBS and incubated with IRDye 800CW anti-rabbit and DRAQ-5 in blocking solution containing 0.2% Tween-20 for 60 minutes at room temperature in the dark. Cells were then washed again six times with 1% Tween-20 in PBS and rinsed twice with PBS. The plates were turned upside down, the residual liquid was absorbed on paper towels and plates were immediately imaged with Odyssey Infrared Imaging Systems. The scanning settings used were medium quality, 169 µm resolution, 3.5 focus offset and intensity setting of 5 for both channels scanned simultaneously. A Janus automated workstation (Perkin Elmer) was used for all liquid handling procedures.

Z′ factor was used to calculate the roubstnes of the screening assay [33]. Briefly, cells on 96 well plates were treated with either 10 µM PHA-767491 or DMSO. Background controls were wells that did not receive either primary antibody nor DRAQ5. Three plates were run on different days and to consider bias due to well location and related systematic errors, the position of the controls was alternated in every run. Each plate was scanned twice with a 180° rotation of the plate. The average intensity values for each well was then calculated based on the two integrated intensity readings generated. Background intensity values were calculated with the same procedure and then subtracted. Finally, pSer40/41MCM2 intensity values were corrected relatively to DRAQ5 intensity in order to account for cell number variability.

For the screening of compound libraries, in order to allow the comparison of measurements across many plates, the relative intensity values for pSer40/41MCM2 were normalized against DMSO in each plate and presented as percentage of inhibition. Wells located at the corners of the 96-well plates were always excluded from the analysis.

Overall average and standard deviation of the percentages of pSer40/41inhibition calculated was 95.1 and 8.9 respectively. A hit compound was defined as a compound that generated a percentage of pSer40/41MCM2 inhibition ≥3.0 overall standard deviations from the overall mean (i.e. 95.1%±26.7%).

## Results

### Validation of novel pSer40/41MCM2 antibody

**Figure 1:**
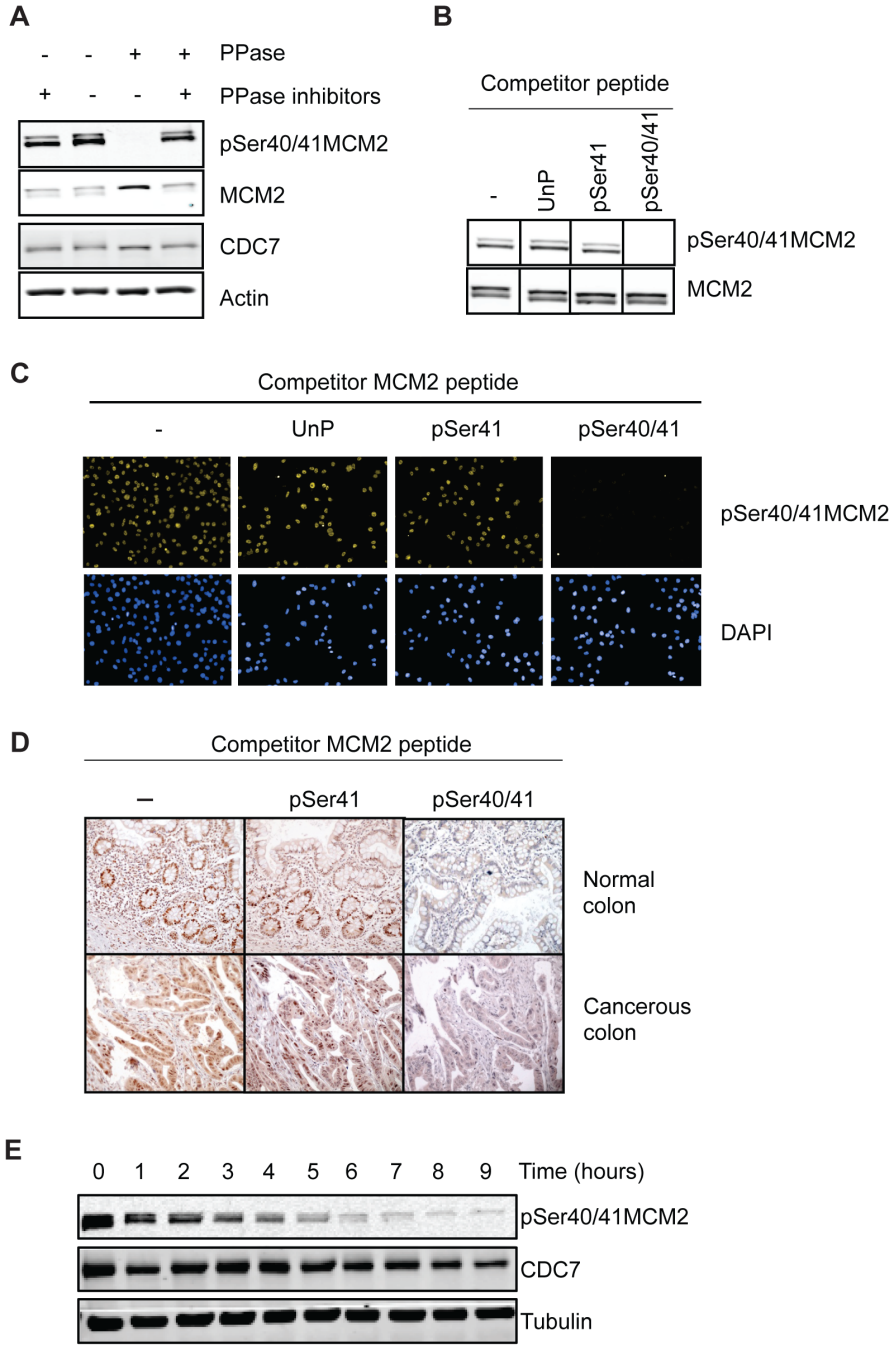
Characterization of pSer40/41MCM2 antibody. A) HeLa whole cell extract was incubated with Lambda phosphatase in the presence or absence of phosphatase inhibitors. Proteins were analyzed by western blot with the indicated antibodies. B) Equal amounts of protein extract were separated on a single SDS-PAGE gel and transferred onto membranes. Vertical slices of membrane (each with identical protein content) were then incubated with anti-pSer40/41MCM2 antibody in the presence of the indicated competitor peptides (upper panels). Membranes were then re-probed with an anti-MCM2 antibody (lower panels). Identical exposures are shown. C) HeLa cells growing on coverslips were fixed and stained with anti-pSer40/41MCM2 antibody in the presence of the indicated competitor peptides. DNA was counterstained with DAPI. D) Serial sections of normal and cancerous colon tissue were used in IHC with the pSer40/41MCM2 antibody in the presence or absence of competitor peptide. Nuclear positivity is shown by brown color. E) HeLa cells were treated with PHA-767491 for the indicated time. Protein extracts were prepared and analysed by western blot with the indicated antibodies.

To assess the effects of compounds on cellular CDC7 kinase activity, we designed a high-throughput in-cell western approach to detect a CDC7-catalysed phosphorylation event. As a specific readout of CDC7 activity we monitored phosphorylation of MCM2 at Serine 40. The residue immediately downstream of Ser40 (Ser41) is constitutively phosphorylated and acts as a priming event for CDC7 phosphorylation on Ser40 [8], [9]. Thus an antibody recognizing double phosphorylated MCM2 on Ser40/41 but not singly phosphorylated pSer41 MCM2 can be used to monitor CDC7-dependent phosphorylation of MCM2 activity [9]. The characterization of a novel pSer40/41MCM2 antibody used in this study is shown in Figure 1. By western blot the antibody recognized two bands of approximately 110 kDa, consistent with the differentially phosphorylated MCM2 isoforms (Figure 1A). The antibody was specific for a phospho-epitope as it failed to recognize MCM2 in HeLa cell extracts treated with lambda phosphatase (Figure 1A). Peptide competition experiments using peptides derived from the MCM2 sequence were then performed to confirm the identity of the phospho-epitope recognized. While competition with an un-phosphorylated peptide or with a mono-phosphorylated peptide at pSer41 did not reduce the immunological reaction, it was instead completely ablated by a doubly phosphorylated pSer40/pSer41 peptide (Figure 1B). The specificity of the antibody was also tested in immunofluorescence and immunohistochemistry experiments. Again in both cases, competition with the doubly-phosphorylated peptide abolishes the majority of the signal detected in tissue sections, whereas competition with unphosphorylated or mono-phosphorylated Ser41 peptide does not (Figure 1C and 1D).

Then, HeLa cells were treated with PHA-767491, the first described CDC7 inhibitor [10], [34] in a time course experiment and protein samples were analysed by western blot. A time-dependent decrease of the signal detected by our antibody was observed, consistent with the antibody detecting pSer40/pSer41 MCM2 and consistent with previous results obtained with a different antibody raised against a similar epitope (Figure 1E) [10], [35]. Taken together, these results show that the new antibody specifically recognizes MCM2 that is phosphorylated on both Ser40 and Ser41. It does not recognize monophosphorylated pSer41MCM2 alone (which is constitutively phosphorylated) but only detects MCM2 when the CDC7-dependent Ser40 is also phosphorylated. This antibody is therefore a suitable reagent for assessing CDC7 activity in cell-based assays.

### Identification of small molecules affecting CDC7 kinase activity *in vivo*

In order to identify compounds affecting pSer40/41 MCM2 phosphorylation in cells, we used in-cell western technology as an antibody-based technique suitable for high-throughput screening of cells [36]. Briefly cells were plated onto 96 well plates, treated with compound, fixed and sequentially incubated with the pSer40/41 MCM2 primary antibody and near-infrared fluorescent dye conjugated secondary antibodies. Immunoreactions were detected and the average intensity of the signal in each well was quantified using a near-infrared imaging system. As pSer40/41 MCM2 signal is also dependent on the number of cells seeded and thus the amount MCM2 protein in each well, counterstaining with either an anti-MCM2 antibody or overall DNA with DRAQ5 was included for normalization purposes.

During the optimization of the assay, the following parameters were first considered: the number of cells seeded per well, the number of washes following staining with the antibody, and the focus offset used during image acquisition. PHA-767491 was used as positive control and as a tool to define the length of treatment required to observe a measurable decrease in pSer40/41MCM2 under these experimental conditions (Figure S1). Robustness of the assay under each condition was evaluated by calculating Z′ factor values. In summary, we found that nine hours of treatment and normalization of cell number using DRAQ5 resulted in the highest Z′ factor value of 0.64, indicating that under these conditions the assay was sufficiently robust to perform a high throughput screen (HTS).

**Figure 2:**
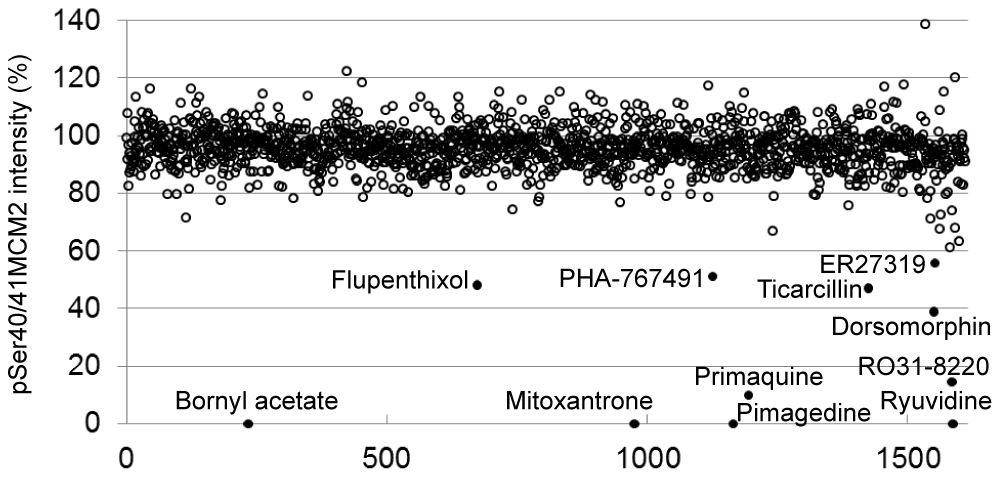
Screening for modulators of Cdc7-dependent phosphorylation of Ser40/41 MCM2. Results of the screening of the Johns Hopkins Clinical Compound Library and Tocris kinase inhibitor library using In Cell Western Technology. Each dot represents a compound and the normalized pSer40/41 MCM2 intensity is reported - See text for experimental details. The names of the hits producing the strongest reduction in phosphorylation at Ser40/41MCM2 are indicated.

The Johns Hopkins Clinical Compounds Library consisting of approximately 1500 drugs [37] and a small library of 80 partially characterized kinase inhibitors were then screened at a concentration of 10 micromolar. A hit compound was defined as one that changed pSer40/41MCM2 phosphorylation levels by more than three standard deviations. Using these criteria, fourteen compounds that decreased and two compounds that increased pSer40/41MCM2 levels were identified (Figure 2 and Table S1).

### Hit confirmation and characterization

**Figure 3:**
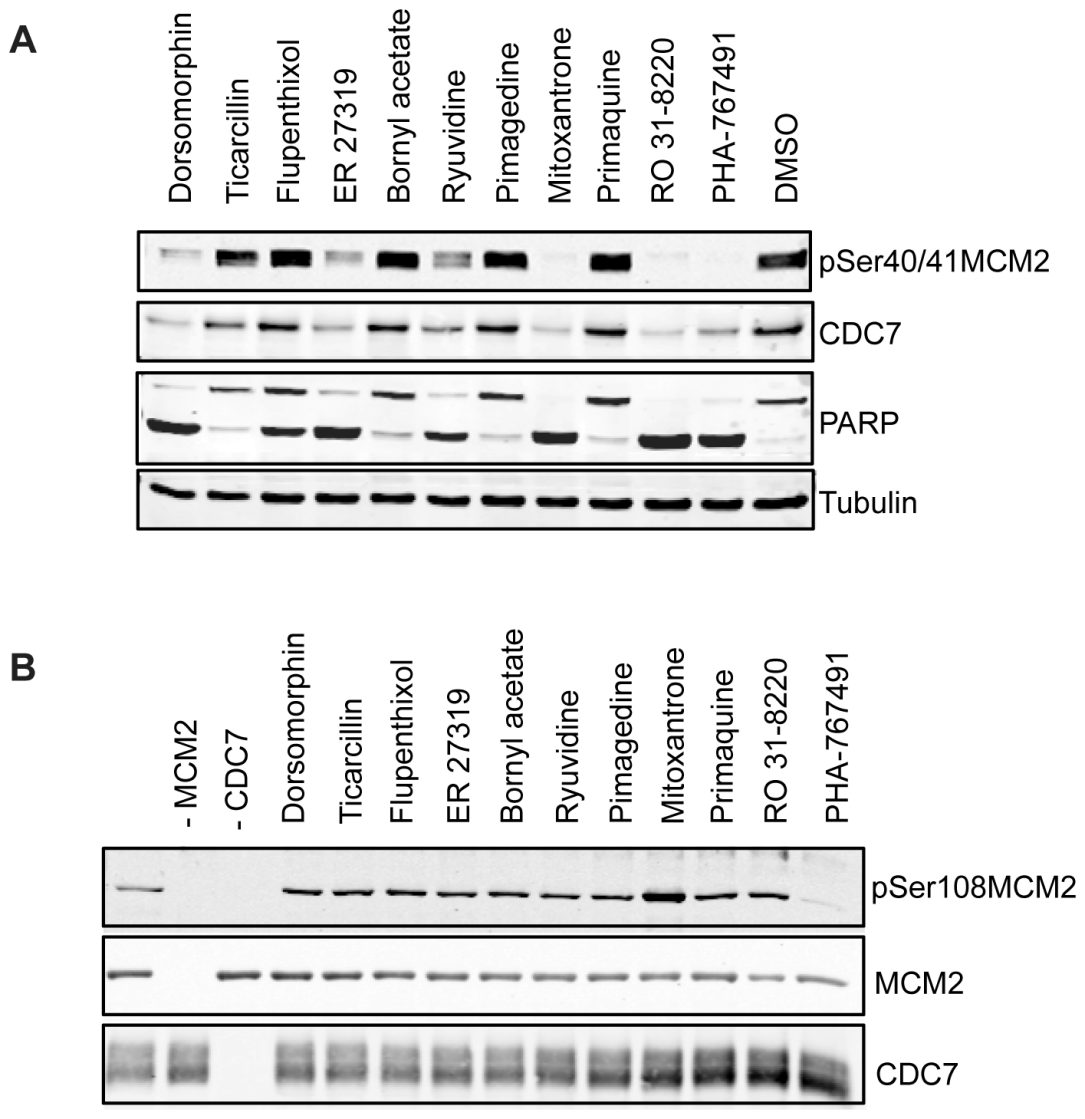
Confirmation and *in vitro* characterization of Hit compounds. A) HeLa cells were treated with the ten compounds that produced the strongest reduction in pSer40/41MCM2 levels in the primary screen. Protein extracts were prepared and levels of residual pSer40/41MCM2 phosphorylation were measured by western blot analysis with the indicated antibodies. B) The same compounds were tested for their ability to inhibit CDC7 kinase activity in *in vitro* kinase assay. Kinase reactions were performed on a synthetic MCM2 substrate in the absence or presence of the indicated drug and reactions were run on SDS-PAGE gels. CDC7 activity on the synthetic MCM2 substrate was monitored by Western blotting with an anti-pSer108MCM2 antibody. As a control the levels of CDC7 kinase and synthetic MCM2 substrate present in each reaction were also assessed and shown to be similar in all reactions.

The ten compounds that caused the strongest decrease in MCM2 pSer40/41 signal were selected for confirmation. HeLa cells were again treated with these compounds, most of them freshly purchased, at 10 µM for nine hours, protein extracts prepared and pSer40/41MCM2 phosphorylation levels were detected by semi-quantitatve western blot. We observed that five out of ten compounds again caused a strong reduction of pSer40/41MCM2 levels, caused a decrease in CDC7 protein levels, and elicited an apoptotic response evident by cleavage of PARP (Figure 3A and Table S1). The treatment of HeLa cells with the other five compounds only very partially reduced pSer40/41 phosphorylation in this assay. The discrepancies between the results obtained for these compounds by the in-cell western primary screen and semi-quantitative western blot reconfirmation are very likely due to differences in either the concentration or possibly in the identity of the compounds in the screening plates and were not further investigated.

We next asked whether any of the drugs identified in the screen was a direct inhibitor of CDC7 kinase by performing *in vitro* kinase assays. Recombinant CDC7/DBF4 kinase was incubated with a GST-MCM2 N-terminal fusion protein and cold ATP. While efficient phosphorylation of MCM2 at Ser40 by CDC7 requires pre-phosphorylation of Ser41 by a different kinase, CDC7 efficiently phosphorylates *in vitro* MCM2 at serine 108 in the absence of any other enzymatic activity [9]. Thus, CDC7 kinase activity *in vitro* was assessed by western blotting using an antibody that recognizes MCM2 phosphorylated at Serine 108. We found that none of the compounds tested inhibited CDC7 kinase activity in this assay (Figure 3B) suggesting that, unlike PHA-767491, these are not direct CDC7 kinase inhibitors *in vitro* but instead could modulate either CDC7 kinase level or its activity in cells.

### Ryuvidine and Mitoxantrone reduce CDC7 levels and block DNA synthesis

**Figure 4:**
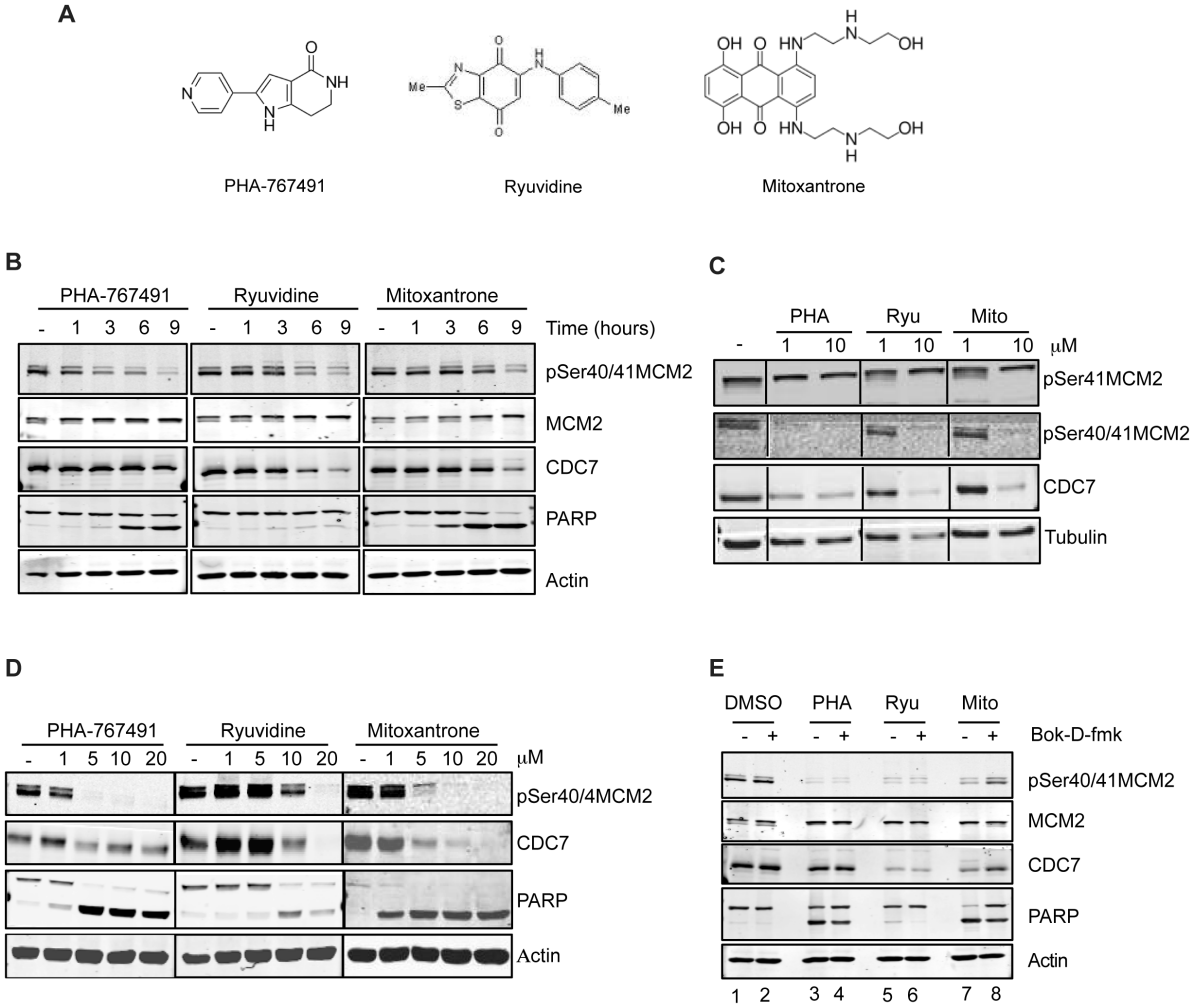
Ryuvidine and Mitoxantrone reduce CDC7 levels and pSer40/41 MCM2 phosphorylation. A) Molecular structure of PHA-767491, Ryuvidine and Mitoxantrone. B) HeLa cells were incubated with either CDC7 inhibitor PHA-767491, Ryuvidine or Mitoxantrone at 10 micromolar each for the indicated time. Protein extracts were prepared and analyzed by western blot with the indicated antibodies. C) Levels of Cdc7-independent pSer41MCM2 and Cdc7-dependent pSer40/41MCM2 phosphorylation assessed in HeLa cells treated with the indicated concentration of PHA-767491, Ryuvidine or Mitoxantrone. D) Cells were incubated with either PHA-767491, Ryuvidine or Mitoxantrone at the indicated concentration for nine hours. Protein extracts were then prepared and analyzed by western blot with the indicated antibodies. E) HeLa cells incubated with either PHA-767491, Ryuvidine or Mitoxantrone at 10 micromolar each for nine hours in the presence or absence of 20 micromolar of caspase inhibitor Boc-D-fmk. Levels of Ser40/41MCM2 phosphorylation, CDC7 and MCM2 abundance as well as PARP cleavage, were assessed by western blotting.

Intriguingly Ryuvidine, a poorly characterized benzothiazole derivative present in the kinase inhibitor library screened, was one of compounds identified as a potential modulator of CDC7 activity (Figure 4A). Ryuvidine was previously described as a kinase inhibitor with specificity against CDK4 [38]–[40], a kinase controlling G1 progression and entry into S-phase of the cell cycle [41], suggesting a possible regulatory or functional link between CDK4 and CDC7 kinases. Thus cellular responses to Ryuvidine were assessed in time- and dose- dependency experiments and compared to the cellular response to the CDC7 inhibitor PHA-767491 and to Mitoxantrone, a Topoisomerase 2 inhibitor that is widely used in chemotherapy [42], that was also identified in the screen.

Firstly, we treated cells with 10 µM of each compound and in time course experiments we observed that, upon Ryuvidine or Mitoxantrone treatment, the decrease of pSer40/41 MCM2 occurred at approximately the same time as a marked decrease in CDC7 protein levels (Figure 4B). Importantly, the reduced MCM2 phosphorylation appeared to be restricted to a CDC7-dependent phosphosite and did not obviously affect the adjacent CDC7–independent phosphorylation of Ser41 (Figure 4C). When cells were incubated with increasing concentrations of the drugs for nine hours, we found that 20 µM Ryuvidine was required for complete dephosphorylation of MCM2 while 5 µM PHA-767491 or Mitoxantrone were sufficient to achieve the same effect (Figure 4D).

All three compounds at active doses induced PARP cleavage, although Ryuvidine to a much lesser extent than PHA-767491 and Mitoxantrone, suggesting that activation of caspases could be involved in both the reduction of CDC7 levels and as a consequence of this, MCM2 phosphorylation levels. To test this hypothesis, HeLa cells were again challenged with the three compounds in the presence or absence of the broad caspase inhibitor Boc-D-fmk [43]. Under these experimental conditions, Boc-D-fmk partially reduced PARP cleavage caused by all the three compounds but, importantly, it rescued Mitoxantrone-induced degradation of CDC7 and pSer40MCM2 de-phosphorylation (Figure 4E lanes 7 and 8).

Altogether these data indicate that decrease of cellular CDC7 kinase levels is the likely reason for the loss of Ser40/41 MCM2 phosphorylation by Ryuvidine and Mitoxantrone and that a Boc-D-fmk sensitive caspase is involved in Mitoxantrone but not in Ryuvidine-induced loss of CDC7 protein.

**Figure 5:**
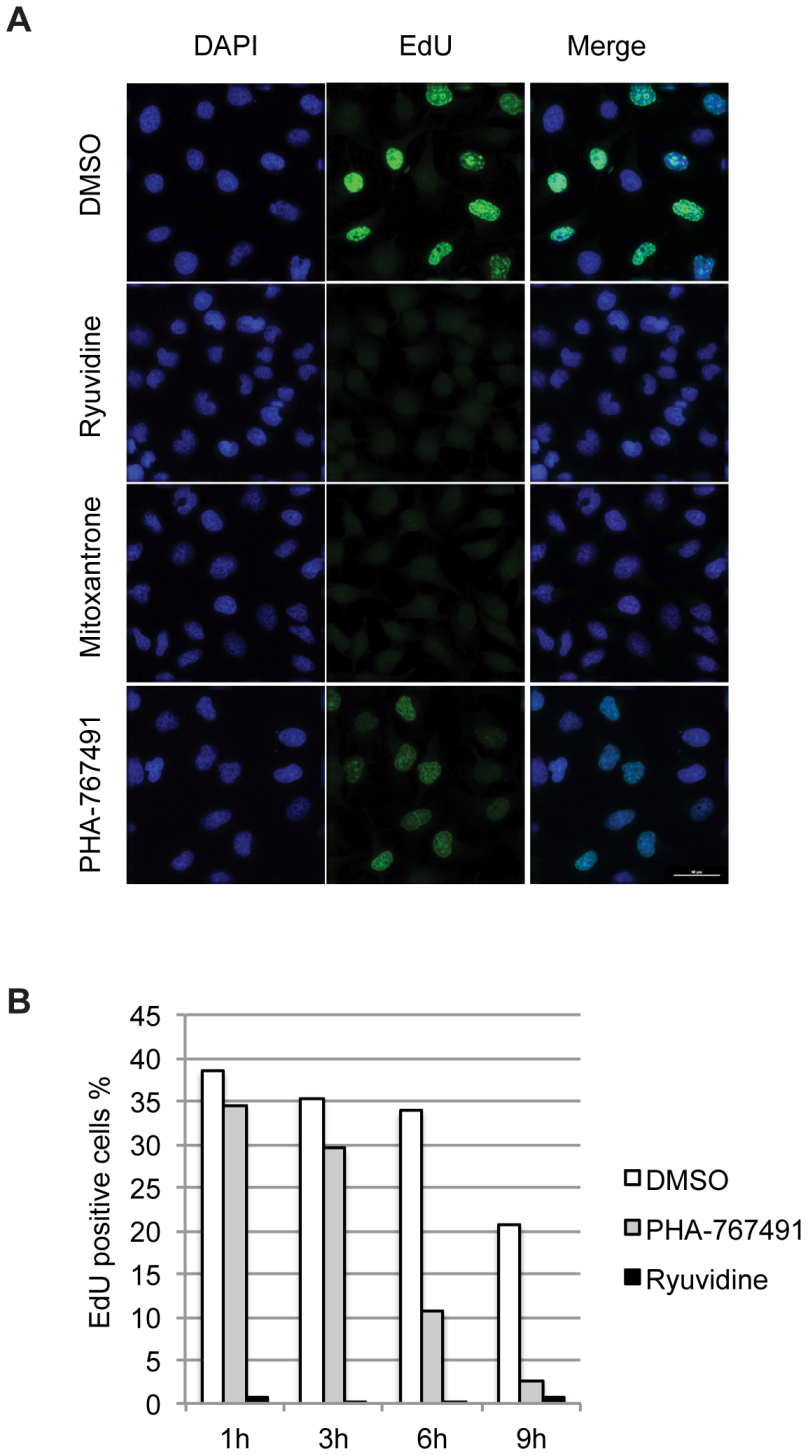
Ryuvidine and Mitoxantrone block DNA synthesis. A) HeLa cells growing on coverslips were incubated for one hour with either Ryuvidine, Mitoxantrone or PHA-767491. 15 minutes before the end of treatment EdU was added and cells were fixed. DNA synthesis was revealed by covalently linking 6-carboxyfluorescein TEG-Azide to incorporated EdU and fluorescence microscopy. Nuclei were stained with DAPI. Representative fields are shown. B) HeLa cells were either mock treated (DMSO) or treated for the indicated times with PHA-767491 or Ryuvidine. Fifteen minutes before the end of treatment EdU was added to the medium. DNA synthesis was analyzed by EdU incorporation assay and Flow Cytometry. Percentage of EdU positive cells at the indicated times after addition of the drugs is reported.

We next investigated whether these compounds affected DNA replication by measuring EdU incorporation in HeLa cells. Cells growing on glass slides were treated with the drugs for different time periods, and after EdU incorporation, DNA synthesis was visualized by fluorescence microscopy. Mitoxantrone, as expected for a known topoisomerase inhibitor, caused an early and complete block of DNA replication. However, surprisingly, we found that 10 µM Ryuvidine also caused a complete block of replication as early as one hour after treatment (Figure 5).

The rapid DNA synthesis inhibition by Ryuvidine, a reported CDK4 kinase inhibitor, was so unexpected that we firstly reconfirmed the findings and kinetics in different cell lines including U2OS osteosarcoma cells and hTERT-immortalized normal human Foreskin Fibroblasts (Figure S2), and secondly, we directly compared it to the effects of PHA-767491. Indeed the kinetics of DNA synthesis inhibition by Ryuvidine and PHA-767491 were very different, as the CDC7 inhibitor PHA-767491 only partially reduced DNA synthesis after one hour (Figure 5A bottom panel), while six to nine hours of treatment with CDC7 inhibitor PHA-767491 was required to completely stop replication in HeLa cells (Figure 5B and [10]).

The fast kinetics of the DNA replication blockade indicate that Ryuvidine is a potent inhibitor of DNA synthesis, however, the observations that Ryuvidine does not directly inhibit CDC7 and that CDC7 protein and activity remain detectable in cells treated with Ryuvidine while DNA synthesis is blocked, suggests that Ryuvidine's effect on DNA synthesis is likely via a mechanism that does not rely on its ability to decrease CDC7 levels or activity.

### Ryuvidine elicits a DNA damage response

In order to further characterize the effects of Ryuvidine on DNA replication we analyzed cellular responses to this drug, and in particular we asked if the replication stress and DNA damage-responsive kinases CHK1, CHK2 and ATM were activated at early times upon addition of this drug. For comparison Mitoxantrone was also included in this analysis as a known agent causing DNA damage [42].

**Figure 6:**
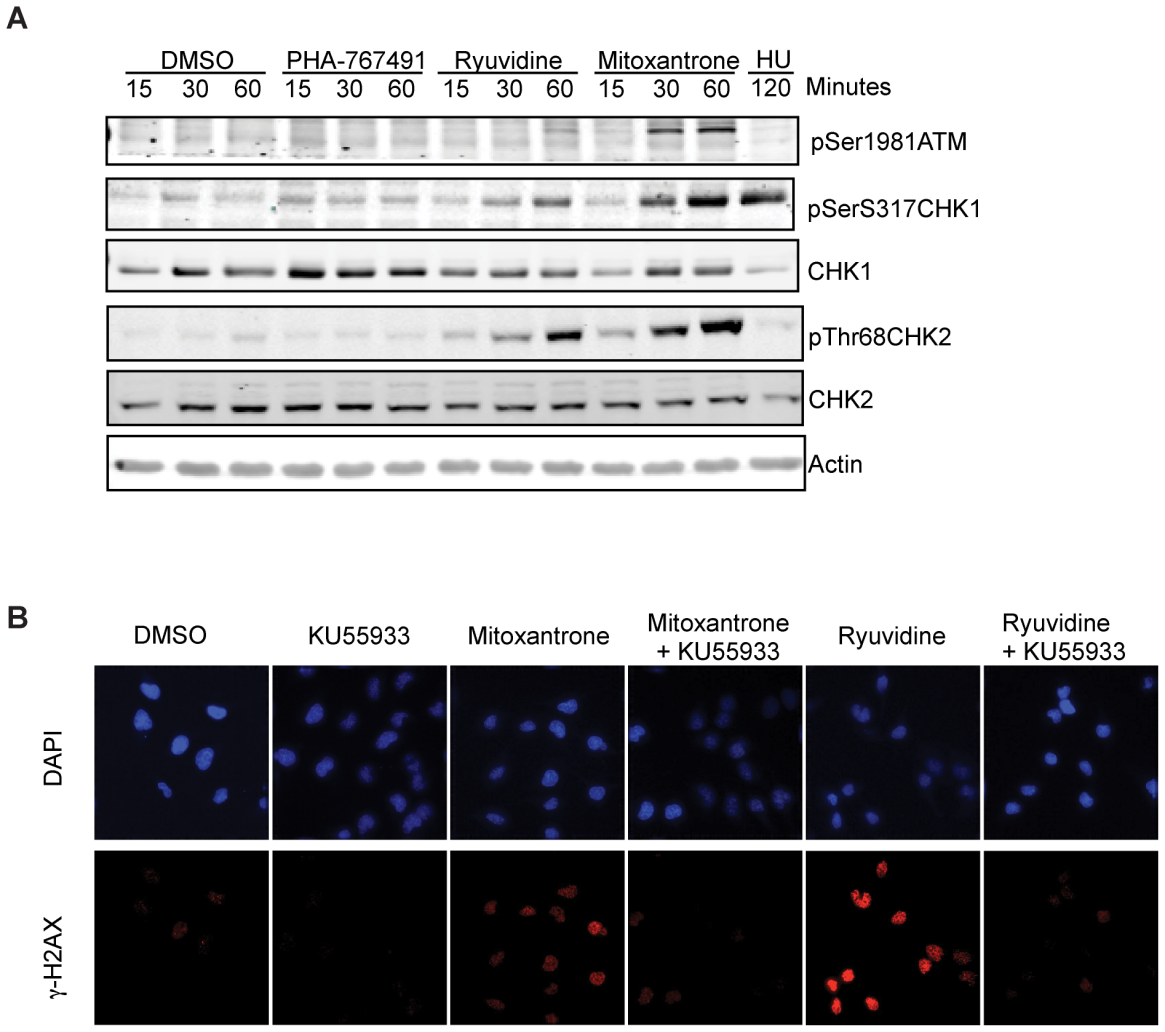
Ryuvidine elicits a DNA damage response. A) Protein extracts prepared from HeLa cells treated with either PHA-767491, Ryuvidine, Mitoxantrone or hydroxyurea (HU) for the indicated time were analyzed by western blot with indicated antibodies. B) Formation of γ-H2AXnuclear foci in cells treated with ATM inhibitor KU55933, Mitoxantrone, Ryuvidine alone or in combination was assessed by fluorescence microscopy. Nuclei were stained with DAPI. Representative fields are shown.

Figure 6A shows that in Ryuvidine treated cells, CHK2 phosphorylation at threonine 68, an ATM-dependent phosphorylation site [44], was strongly induced and ATM auto-phosphorylation at Ser1981 was detected, although at a later time. CHK1 phosphorylation at Ser317, a site that can be phosphorylated by both ATM and ATR [45], [46] was also induced. In these assays, a two-hour treatment with the ribonucleotide reductase inhibitor Hydroxyurea (HU) only caused CHK1 phosphorylation. Mitoxantrone, consistent with its known mechanism of action as a topoisomerase inhibitor, caused robust phosphorylation of all the kinases tested, while PHA767491, as previously reported [5], [10] did not activate a checkpoint response (Figure 6A).

To further confirm a role for ATM in the response to Ryuvidine, treated cells were stained with an antibody against pSer139H2AX, also known as γ-H2AX, an established marker of double-stranded DNA breaks [47], [48]. We observed that Ryuvidine treated cells, similar to Mitoxantrone treated cells, displayed strong nuclear positivity (Figure 6B). Importantly, co-treatment with a specific ATM inhibitor, KU55933, prevented γ-H2AXformation (Figure 6B) although it did not restore DNA synthesis (data not shown).

Thus we conclude that Ryuvidine likely causes double-stranded DNA breaks and activates the ATM- CHK2 response.

## Discussion

DNA replication is a well-established target mechanism of many anticancer drugs, which, either directly or indirectly, affect the processivity of ongoing replication forks and cause a DNA damage response. More recently kinases that promote the initiation of DNA replication such as cyclin-dependent kinases and CDC7 kinase have been proposed as novel targets for drug discovery in Oncology [21], [22]. In the current study, the availability of a large quantity of an antibody that specifically detected CDC7-dependent phosphorylation on MCM2, enabled us to develop a robust cell-based assay to identify modulators of CDC7 activity and this methodology was optimized for use in a high through-put screen.

We screened a limited collection of generic kinase inhibitors and FDA approved drugs and identified and reconfirmed several compounds that alter cellular levels of pSer40/41MCM2 in HeLa cells. While none of these drugs appear to directly inhibit CDC7 kinase in biochemical assays, we have noted that, in general, pSer40/41MCM2 levels closely mirror CDC7 levels, and that many of the hit compounds affect intracellular CDC7 kinase levels. As multiple regulatory mechanisms contribute to controlling the level of CDC7, further mechanistic studies will be required to determine how these compounds affect CDC7 aboundance.

From our screening, two compounds, Mitoxantrone and Ryuvidine, were selected and further characterized. Mitoxantrone is an anthracycline compound related to Doxorubicin, and is currently used as a chemotherapeutic agent against a range of cancers [42], [49]. Like Doxorubicin, Mitoxantrone is a topoisomerase II poison and results in the formation of DNA double-strand breaks [50], [51]. Treatment of cells with Mitoxantrone induces a DNA DSB response as detected by ATM phosphorylation [52] and γ-H2AXformation [53]. All these findings were reconfirmed in our analysis. Surprisingly, Mitoxantrone has recently also been found to be a potent inhibitor of the PIM1 kinase by binding within the ATP pocket [54]. Intriguingly, in our *in vitro* kinase assay, we observed a reproducible stimulation of CDC7 activity by Mitoxantrone, reminiscent of the weak stimulation also observed on AKT1 kinase [54]. The mechanisms underlying Mitoxantrone-induced reduction of pSer40/41MCM2 and CDC7 levels in cells, are likely related to a caspase-dependent degradation of either CDC7 itself, or degradation of a different protein that controls CDC7 stability, since both events are antagonized by a generic caspase inhibitor. To date, it is not clear if CDC7 destabilization is a general feature of apoptotic cell death or a specific event caused by Mitoxantrone, since it is not observed in PHA-767491 induced apoptosis and other anthracyclines such as Doxorubicin, Daunorubicin, Idarubicin, and Epirubicin, that were included in our in-cell western screen did not cause a decrease in pSer40/41MCM2 levels. Time course experiments suggest that the rapid shut down of DNA synthesis and formation of γ-H2AXfoci by Mitoxantrone is most likely due to concomitant binding to DNA and its inhibition of topoisomerases, which impedes progression of replication and transcription forks, leading to DNA breaks and the strong activation of DNA damage and DNA replication checkpoints [55] and is not due to its effect on CDC7.

More interestingly, we found that Ryuvidine, developed as a kinase inhibitor with reported specificity against CDK4 by Ryu and coworkers [38], also reduced the levels of pSer40/41MCM2, decreased CDC7 protein levels and caused a DNA synthesis blockade and activation of a DNA damage response with kinetics that are difficult to attribute to either CDC7 or CDK4 inhibition. Although we cannot rule out that decrease of CDC7 protein is the secondary effect of loss of cell viability, this process appears to be independent from caspase activity.

Ryuvidine was shown to cause cell death in a panel of cancer cell lines [38] and this cytotoxic activity is also present in cell lines, such as HeLa cells that lack a functional Rb pathway, suggesting that the main mechanism of action of Ryuvidine may not be related to CDK4 inhibition. More recent data from high throughput experiments raise questions about whether Ryuvidine can be considered a direct kinase inhibitor [56], [57]. Federov and coworkers assessed binding of 156 kinase inhibitor compounds to a panel of 60 recombinant human kinases by thermal stability shift experiments, and Ryuvidine was not found to alter the thermal stability of any of the kinases tested [56]. Similarly Anastassiadis and coworkers assessed the activity of 178 kinase inhibitors against 300 recombinant human protein kinases and Ryuvidine was not found to significantly inhibit any of the kinases tested, including CDK4 [57].

From a chemical perspective Ryuvidine is a 4,7-benzothiazoledione derivative, which could combine the properties of benzothiazole and *p*-benzoquinone. Benzothiazoles are heterocyclic compounds that resemble DNA bases and several of these compounds have been studied for their anticancer activity [58], [59]. More importantly, quinone groups, which are common in several natural products, have been extensively reported to generate DNA damage [60], [61] by two possible mechanisms: direct alkylation of DNA bases and oxidative damage by production of reactive oxygen species [62].

Our analysis shows that Ryuvidine elicits a strong ATM-dependent DNA damage response suggesting that direct binding to DNA and/or formation of double strand breaks is the main cause of its anti-proliferative and cytotoxic activity. However, our study also showed that Ryuvidine causes a decrease in CDC7 protein levels and, although the mechanism by which this occurs remains to be elucidated, it has been reported that down-regulation of CDC7 in the presence of genotoxic drugs increases the number of cells that undergo apoptosis [63]. Therefore, it is possible that both the ability of Ryuvidine to cause DNA damage as well as reducing CDC7 levels contribute to its cytotoxic activity. Interestingly, CDC7 has also recently been shown to phosphorylate and stabilize Tob, an anti-apoptotic factor, suggesting that CDC7 contributes to prosurvival signaling and maintaining the viability of cells with damaged DNA [64].

In summary, in an effort to identify small molecules that could be rescued or repurposed as CDC7 inhibitors, we set up an “in-cell western” assay suitable for high-throughput screening. We used this assay to identify potential CDC7 inhibitors present in the Johns Hopkins Clinical Compounds Library and in a small partially characterized kinase library. We identified two compounds (Ryuvidine and Mitoxantrone) that inhibited CDC7-dependent phosphorylation of MCM2. Analysis of both compounds demonstrated that they were not direct inhibitors of CDC7 kinase but reduced CDC7 protein levels in cells. Both compounds block DNA synthesis but the data suggests that this is due to activation of the DNA damage response rather than a direct effect on CDC7. Finally we demonstrated that Ryuvidine, previously described as a CDK4 inhibitor, induces DNA damage and the DNA damage response, which is likely the main cause of its antiproliferative and cytotoxic activity.

## Supporting Information

Figure S1 **Assay development of an In Cell Western assay.** Different experimental conditions were tested and the robustness of the assay assessed by calculating the Z′ score. In each panel triangles correspond to wells in which cells were mock-treated while diamonds correspond to wells in which cells were treated with PHA-767491. A) Comparison of normalization of pSer40/41MCM2 phosphorylation using either an anti-MCM2 antibody or DNA staining with DRAQ5. B) Comparison of six hours vs nine hours treatment.
(TIF)

Figure S2 **Ryuvidine and Mitoxantrone block DNA replication in Human Foreskin Fibroblasts and U2OS osteosarcoma cells.** Human Foreskin Fibroblasts (HFF) and U2OS cells growing on coverslips were incubated for one hour with either Ryuvidine or Mitoxantrone. 15 minutes before the end of treatment EdU was added and then cells were fixed. DNA synthesis was revealed by covalently linking 6-carboxyfluorescein TEG-Azide to incorporated EdU and fluorescence microscopy. Nuclei were stained with DAPI. Representative fields are shown.
(TIF)

Table S1 **Information on the reported HIT compounds either increasing or decreasing pSer40/41MCM2 in HeLa cells.** Residual levels of MCM2 phosphorylation compared to mock treated cells measured in the primary screening by in cell western or in the reconfirmation by quantitative western blotting (see text for details).
(DOCX)
