# Supplementary figures and images for: A High Through-Put Screen for Small Molecules Modulating MCM2 Phosphorylation Identifies Ryuvidine as an Inducer of the DNA Damage Response
Source: PLoS One. 2014 Jun 5;9(6):e98891. doi: 10.1371/journal.pone.0098891 (PMC4047068; doi:10.1371/journal.pone.0098891)

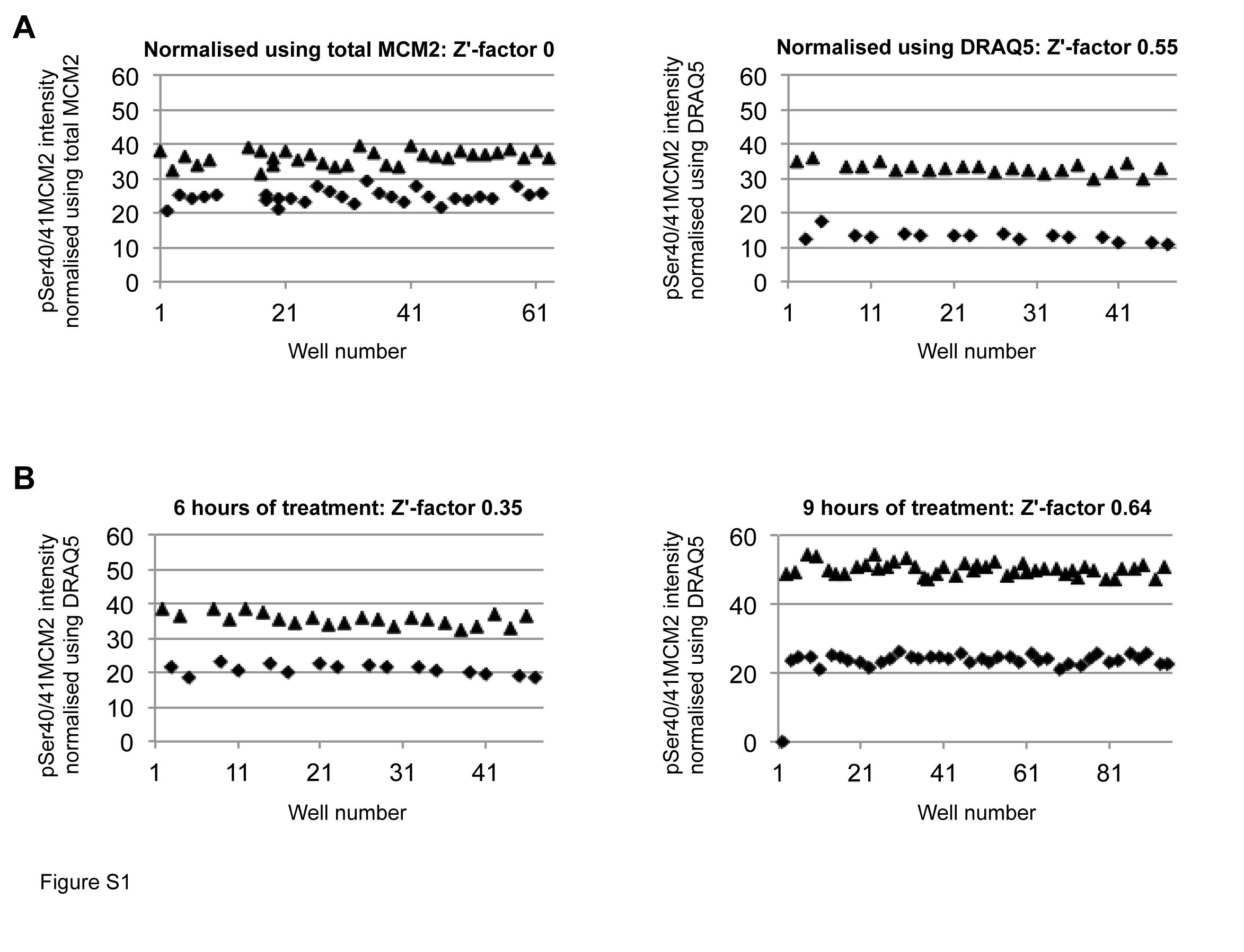

Supplement: Figure S1 — Assay development of an In Cell Western assay. Different experimental conditions were tested and the robustness of the assay assessed by calculating the Z′ score. In each panel triangles correspond to wells in which cells were mock-treated while diamonds correspond to wells in which cells were treated with PHA-767491. A) Comparison of normalization of pSer40/41MCM2 phosphorylation using either an anti-MCM2 antibody or DNA staining with DRAQ5. B) Comparison of six hours vs nine hours treatment. (TIF) [file pone.0098891.s001.tif]

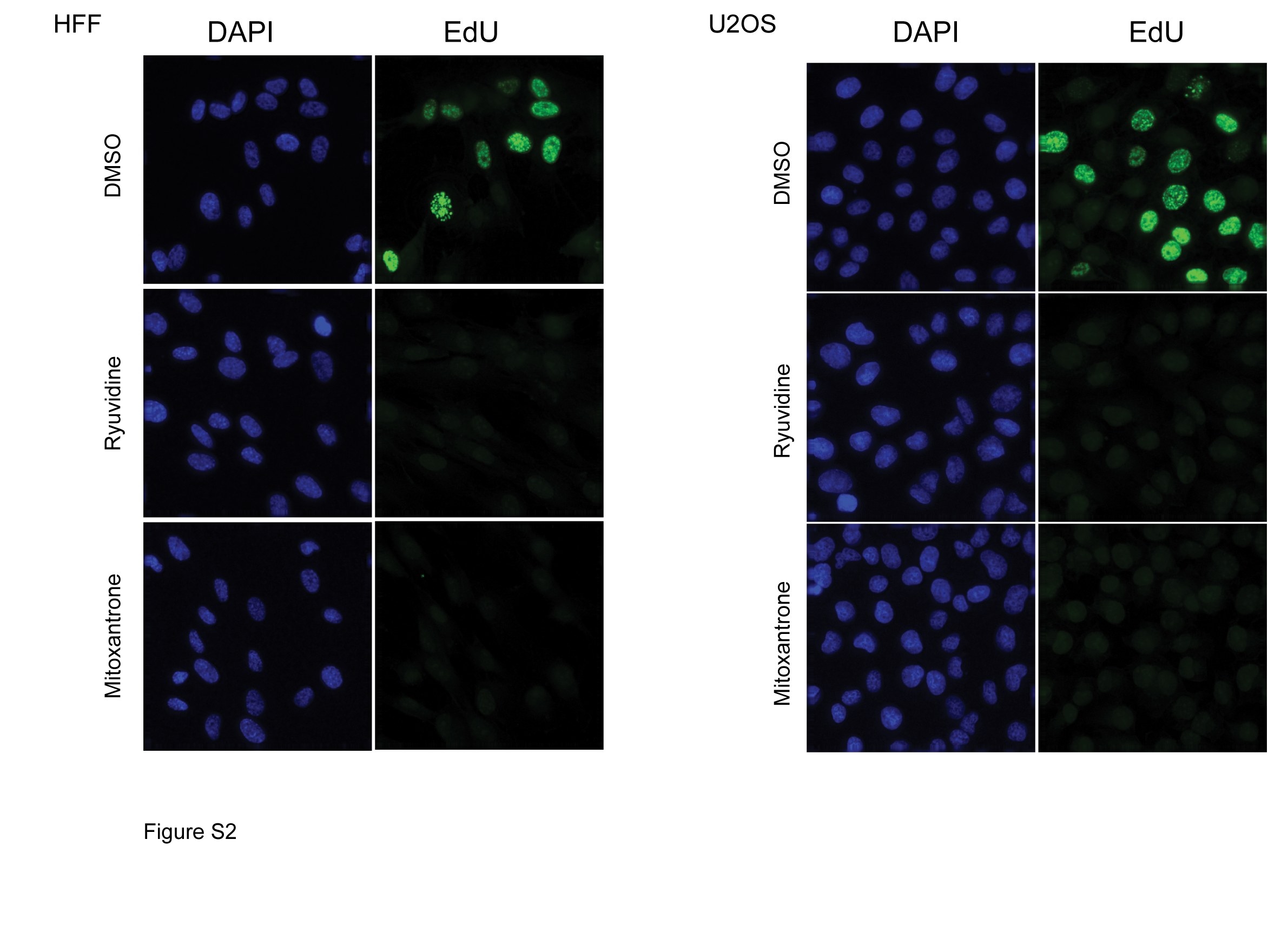

Supplement: Figure S2 — Ryuvidine and Mitoxantrone block DNA replication in Human Foreskin Fibroblasts and U2OS osteosarcoma cells. Human Foreskin Fibroblasts (HFF) and U2OS cells growing on coverslips were incubated for one hour with either Ryuvidine or Mitoxantrone. 15 minutes before the end of treatment EdU was added and then cells were fixed. DNA synthesis was revealed by covalently linking 6-carboxyfluorescein TEG-Azide to incorporated EdU and fluorescence microscopy. Nuclei were stained with DAPI. Representative fields are shown. (TIF) [file pone.0098891.s002.tif]
