# Supplementary material for: A High Through-Put Screen for Small Molecules Modulating MCM2 Phosphorylation Identifies Ryuvidine as an Inducer of the DNA Damage Response
Source: PLoS One. 2014 Jun 5;9(6):e98891. doi: 10.1371/journal.pone.0098891 (PMC4047068; doi:10.1371/journal.pone.0098891)
Supplement: Table S1 — Information on the reported HIT compounds either increasing or decreasing pSer40/41MCM2 in HeLa cells. Residual levels of MCM2 phosphorylation compared to mock treated cells measured in the primary screening by in cell western or in the reconfirmation by quantitative western blotting (see text for details). (DOCX) [file pone.0098891.s003.docx]

**Table S1**

| **Name** | **Functions** | **Library** | **Primary screening by In Cell Western**  **%pSer40/41Mcm2** | **Reconfirmation by quantitative western blot**  **%pSer40/41Mcm2** |
| --- | --- | --- | --- | --- |
| DEBC | Akt inhibitor ([Thimmaiah et al., 2005](#_ENREF_11)) | Tocris | 138.65 | 129.7 |
| Clothiapine | Blocks GABA receptors ([Squires and Saederup, 1998](#_ENREF_10)) | JHCCL | 122.27 | Not tested |
| Quinacrine | Antimalarial treatment ([Chauhan and Srivastava, 2001](#_ENREF_3)) | JHCCL | 66.74 | Not tested |
| IKK16 | IKK kinase inhibitor ([Waelchli et al., 2006](#_ENREF_14)) | Tocris | 67.68 | 55.6 |
| SU4312 | VEGFR and PDGFR kinase inhibitor ([Zaman et al., 1999](#_ENREF_15)) | Tocris | 63.18 | Not tested |
| Purvalanol A | Cdc2, Cdk2, Cdk4 inhibitor ([Villerbu et al., 2002](#_ENREF_13)) | Tocris | 61.27 | 66.4 |
| ER 27319 | Syk kinase inhibitor ([Moriya et al., 1997](#_ENREF_6)) | Tocris | 55.82 | 13.69 |
| Flupentixol | Dopamine receptor antagonist ([Shen et al., 2012](#_ENREF_9)) | JHCCL | 48.15 | 68.9 |
| Ticarcillin | Penicillin derivative ([Brogden et al., 1980](#_ENREF_2)) | JHCCL | 47.06 | 28.9 |
| Dorsomorphin | AMPK inhibitor ([Zhou et al., 2001](#_ENREF_16)) | Tocris | 38.89 | 21.5 |
| RO 31-8220 | Protein kinase C inhibitor, voltage-dependent sodium channel inhibitor ([Lingameneni et al., 2000](#_ENREF_5)) | Tocris | 14.56 | 2.1 |
| Primaquine | Antimalarial treatment ([Baird and Hoffman, 2004](#_ENREF_1)) | JHCCL | 9.94 | 65.7 |
| Mitoxantrone | Topoisomerase II poison ([Pommier et al., 2010](#_ENREF_7)) | JHCCL | 0.0 | 1.6 |
| Pimagedine | Reacts with αeacts with l., 2010F_7" ([Thornalley, 2003](#_ENREF_12)) | JHCCL | 0.0 | 68.1 |
| Bornyl acetate | Antiseptic ([Jeong et al., 2007](#_ENREF_4)) | JHCCL | 0.0 | 56.4 |
| Ryuvidine | Cdk4 inhibitor ([Ryu et al., 2000](#_ENREF_8)) | Tocris | 0.0 | 18.7 |

**References**

Baird, J.K., and Hoffman, S.L. (2004). Primaquine therapy for malaria. Clinical infectious diseases : an official publication of the Infectious Diseases Society of America *39*, 1336-1345.

Brogden, R.N., Heel, R.C., Speight, T.M., and Avery, G.S. (1980). Ticarcillin: a review of its pharmacological properties and therapeutic efficacy. Drugs *20*, 325-352.

Chauhan, P.M., and Srivastava, S.K. (2001). Present trends and future strategy in chemotherapy of malaria. Current medicinal chemistry *8*, 1535-1542.

Jeong, S.I., Lim, J.P., and Jeon, H. (2007). Chemical composition and antibacterial activities of the essential oil from Abies koreana. Phytotherapy research : PTR *21*, 1246-1250.

Lingameneni, R., Vysotskaya, T.N., Duch, D.S., and Hemmings, H.C., Jr. (2000). Inhibition of voltage-dependent sodium channels by Ro 31-8220, a 'specific' protein kinase C inhibitor. FEBS letters *473*, 265-268.

Moriya, K., Rivera, J., Odom, S., Sakuma, Y., Muramato, K., Yoshiuchi, T., Miyamoto, M., and Yamada, K. (1997). ER-27319, an acridone-related compound, inhibits release of antigen-induced allergic mediators from mast cells by selective inhibition of fcepsilon receptor I-mediated activation of Syk. Proceedings of the National Academy of Sciences of the United States of America *94*, 12539-12544.

Pommier, Y., Leo, E., Zhang, H., and Marchand, C. (2010). DNA topoisomerases and their poisoning by anticancer and antibacterial drugs. Chemistry & biology *17*, 421-433.

Ryu, C.K., Kang, H.Y., Lee, S.K., Nam, K.A., Hong, C.Y., Ko, W.G., and Lee, B.H. (2000). 5-Arylamino-2-methyl-4,7-dioxobenzothiazoles as inhibitors of cyclin-dependent kinase 4 and cytotoxic agents. Bioorganic & medicinal chemistry letters *10*, 461-464.

Shen, X., Xia, J., and Adams, C.E. (2012). Flupenthixol versus placebo for schizophrenia. The Cochrane database of systematic reviews *11*, CD009777.

Squires, R.F., and Saederup, E. (1998). Clozapine and several other antipsychotic/antidepressant drugs preferentially block the same 'core' fraction of GABA(A) receptors. Neurochemical research *23*, 1283-1290.

Thimmaiah, K.N., Easton, J.B., Germain, G.S., Morton, C.L., Kamath, S., Buolamwini, J.K., and Houghton, P.J. (2005). Identification of N10-substituted phenoxazines as potent and specific inhibitors of Akt signaling. The Journal of biological chemistry *280*, 31924-31935.

Thornalley, P.J. (2003). Use of aminoguanidine (Pimagedine) to prevent the formation of advanced glycation endproducts. Archives of biochemistry and biophysics *419*, 31-40.

Villerbu, N., Gaben, A.M., Redeuilh, G., and Mester, J. (2002). Cellular effects of purvalanol A: a specific inhibitor of cyclin-dependent kinase activities. International journal of cancer Journal international du cancer *97*, 761-769.

Waelchli, R., Bollbuck, B., Bruns, C., Buhl, T., Eder, J., Feifel, R., Hersperger, R., Janser, P., Revesz, L., Zerwes, H.G.*, et al.* (2006). Design and preparation of 2-benzamido-pyrimidines as inhibitors of IKK. Bioorganic & medicinal chemistry letters *16*, 108-112.

Zaman, G.J., Vink, P.M., van den Doelen, A.A., Veeneman, G.H., and Theunissen, H.J. (1999). Tyrosine kinase activity of purified recombinant cytoplasmic domain of platelet-derived growth factor beta-receptor (beta-PDGFR) and discovery of a novel inhibitor of receptor tyrosine kinases. Biochemical pharmacology *57*, 57-64.

Zhou, G., Myers, R., Li, Y., Chen, Y., Shen, X., Fenyk-Melody, J., Wu, M., Ventre, J., Doebber, T., Fujii, N.*, et al.* (2001). Role of AMP-activated protein kinase in mechanism of metformin action. The Journal of clinical investigation *108*, 1167-1174.
